# Evaluating performance of dental caries detection methods among third-year dental students

**DOI:** 10.1186/1472-6831-13-70

**Published:** 2013-12-06

**Authors:** Heini Parviainen, Hannu Vähänikkilä, Marja-Liisa Laitala, Leo Tjäderhane, Vuokko Anttonen

**Affiliations:** 1 Institute of Dentistry, Department of Cariology, Endodontology and Paedodontics, University of Oulu, Oulu POB 5281 , Finland; 2 University Hospital of Oulu, Oulu, Finland; 3 Institute of Dentistry, Department of Cariology, University of Turku, Turku, Finland

**Keywords:** Caries detection methods, Dental student, ICDAS

## Abstract

**Background:**

Reliable caries detection is a cornerstone in the modern caries treatment schema. This study aimed to evaluate adopting traditional and new caries detection methods by third-year dental students.

**Methods:**

Fifty-seven students were given lectures on caries detection, after which they evaluated 27 extracted carious teeth using traditional clinical assessment (CE), Nyvad’s, and ICDAS methods. On three teeth they also performed DIAGNOdent pen® (LF) scanning. Histological scores of the sectioned teeth (ICDAS, LF) and activity estimations of the lesions by the supervisors were used as golden standards (Nyvad, CE). For the ICDAS method , sensitivity and specificity were calculated using dentine caries (D3) as a cut-off point. Mean ICC and kappa values were calculated to evaluate interexaminer agreement for all lesions and methods. Spearman’s correlation coefficient evaluated LF scanning.

**Results:**

ICDAS method presented good sensitivity **(**0.78) and specificity (0.87). The inter-examiner agreement for different methods was fair or good (CE ICC = 0.69, κ = 0.53; Nyvad’s method ICC = 0.68, κ = 0.48, ICDAS ICC = 0.66, κ = 0.47). Variation in LF values was the greatest with lesions extending to middle third of dentin. In that case, the Spearman’s correlation coefficient was also the weakest.

**Conclusions:**

To follow the guidelines by the European Core Curriculum on Cariology, the third year dental students are introduced to methods for detecting lesion depth and assessing lesion activity as well as using new caries detection methods. Their performance in estimating lesion depth is good, and fair to good in estimating lesion activity even after basic training only.

## Background

The base in the new schema of non-invasive and invasive caries treatments lies on treating dental caries or controlling the course of the disease by choosing the right method of treatment at the right time. The new schema is based on detecting the early signs of the disease. However, it has been shown that for clinical practitioners, detection of caries lesions reliably, and particularly detecting early signs of the disease, is challenging [1,2]. Teaching caries detection, assessment and synthesis remains as a core topic in the dental curriculum. This was also emphasized in the recent European Core Curriculum on Cariology, which was published with an aim to provide a framework of content and goals for education in cariology to undergraduate dental students [3].

With an ideal caries diagnostic method, all caries lesions from the earliest signs to the cavitation stage could be reliably detected and the activity of the lesions assessed. An ideal detection method would also determine the fully sound teeth intact, and the outcome of the detection should be able to be repeated independently of the examiner or the environment. The detection method should be easy to learn, simple to use and applicable to all surfaces of the teeth. Currently, none of the traditional methods completely fulfills these requirements.

Ekstrand et al. [4] were the first ones to introduce a classification of caries lesions connecting the specific clinical signs with histological findings (Table 1) [4]. The ICDAS system (the International Caries Detection and Assessment System) is the most recent effort to create an accurate and reproducible diagnostic tool on the basis of the best available evidence on caries detection methods (Table 2) [5]. The European Core Curriculum in Cariology recommends teaching ICDAS to dental students [6]. The ICDAS method has been shown to be easily adopted by dental students [7,8], with no differences between the faculty and dental students in classifying occlusal caries lesions in vitro [9]. Gimenez et al. [10] in their in vivo study also reported no difference in capability of examiners with different work experience, in detecting caries lesions or assessing their acitivity [10]. Yet, in a recent work by Foley [11] intra- and inter- and intraexaminer reliability in using ICDAS method by dental students was fair [11].

**Table 1:** **Criteria for the scores of the histological caries lesion depth [**[4]**]**

**Score**	**Visual criteria**	**Histological criteria**
**0**	No or slight change in enamel after prolonged air drying	Sound
**1**	Opacity or discoloration hardly visible on wet surface but distinct after air drying	Caries/Radiolucency extending to the outer half of the enamel
**2**	Opacity distinctly visible without air drying	Caries/Radiolucency extending to the inner half of the enamel or the outer third of dentine
**3**	Localized enamel break- down in opaque or discolored enamel and/or grayish discoloration from the under lying dentine	Caries/Radiolucency extending to the middle third of the dentine
**4**	Cavitation in opaque or discolored enamel exposing the dentine	Caries/Radiolucency extending to the inner third of the dentine

These criteria were used as golden standard values of the dissected teeth.

**Table 2:** Criteria for the ICDAS scores (international caries detection and assessment system coordinating committee 2005b)

**Score**	**ICDAS criteria**
**0**	Sound
**1**	First visual change in enamel (seen only after prolonged air drying or restricted to within the confines of a pit or fissure)
**2**	Distinct visual change in enamel
**3**	Localized enamel breakdown (without clinical visual signs of dentinal involvement)
**4**	Underlying dark shadow from dentine
**5**	Distinct cavity with visible dentine
**6**	Extensive distinct cavity with visible dentine

To assess caries lesion activity in addition to lesion depth, e.g. Nyvad’s method as well ICDAS-LAA-method have been introduced (Table 3). In both of these methods the activity of the lesion is based on the colour and texture of the tooth surface considering plaque stagnation. Nyvad’s method can be called descriptive, whereas ICDAS-LAA is prescriptive i.e. cilinical signs of activity are given numeric values and the sum score indicates lesion activity [12]. In the health centers in Finland, the activity of initial lesions has been considered and registered for decades (CE), yet in the method, the decision is made without specific criteria (descriptive). This protocol has been used in earlier studies and changes in clinical stauts have been reported being well associated with i.e. change in laser fluorescence values over time [13].

**Table 3:** **Criteria for a modified version of the scores by Nyvad et al.**[12]**; scores 7–9, which relate to restored surfaces are omitted**

**Score**	**Modified Nyvad**
0	Sound
1	Active caries (intact surface)
2	Inactive caries (intact surface)
3	Active caries (surface discontinuity)
4	Inactive caries (surface discontinuity)
5	Active caries (cavity)
6	Inactive caries (cavity)

In the recent two decades, numerous new quantitative caries detection methods have been introduced to improve especially early detection of caries lesions. Teaching these methods is not part of the main core of the curriculum for dental students, but the students should be aware of them [3]. One of the most popular, and also clinically used, additional method is laser fluorescence or LF (DIAGNOdent®, *KaVo* Dental GmbH, Biberach, Germany) introduced two decades ago [14]. The laser fluorescence method has been found to possess good sensitivity and reproducibility. The method has been recommended to be used as an adjunct to visual inspection [15].

Because caries detection is a corner stone in controlling caries and core I in dental curriculum, the aim in this study was to investigate how easily caries detection and activity assessment are adopted by dental students previously unfamiliar with caries diagnostics. Another aim was to investigate how accurate their performance is using different methods after basic training. The methods used here, were ICDAS, Nyvad’s method, clinical estimation protocol used widely in Finland (CE) and laser fluorescence (LF). Teaching was done by combining introductory lectures and an *in vitro* hands-on workshop. It was hypothesized that the accuracy of dentine caries lesion detection by third year dental students using the ICDAS method would be good. It was also hypothesized that reproducibility i. e. inter-examiner agreement between the students would be fair to good for using different caries detection and lesion activity assessment methods.

## Methods

### Subjects

To teach caries detection, the third-year dental students (n = 57) in the Institute of Dentistry, University of Oulu, Finland, had three hours of lectures followed by a hands-on course in a simulation laboratory. The lectures comprised an introduction to caries disease and its clinical manifestations, progression and arrest of the lesions as well as clinical and histological findings at different stages. Lesion depth and respective histological changes as well as lesion activity were illustrated by clinical photographs of teeth (whole and sectioned). The students were also introduced to lesion depth according to clinical signs and different caries detection systems and the criteria there of (Tables 1, 2 and 3). The students had no prior clinical experience and most of them had never before seen a carious tooth.

### Study material

After the theoretical part, caries detection was practiced on extracted teeth in the hands-on session. A sample of 27 permanent molars was collected of the pool of extracted teeth donated for research purposes to the Institute of Dentistry, University of Oulu, Finland. All the teeth had been stored in denatured alcohol after the extraction and were only removed for the time of the evaluation. The teeth were selected by two supervisors (HP and VA). For the assessment session, the teeth were placed in numbered plastic containers (1–27).

### Ethics

No donors of the study material could be identified. According to The Medical Research Act (No. 488/1999), no ethical evaluation is required for non-interventional studies. Therefore the present study did not require a statement from the Ethics Committee.

### Examination protocol

The students were working in teams of two, but each student was advised to record his/her own assessment manually on an evaluation sheet. During the workshop, the students were given a written document on the criteria of the detection methods (Tables 1, 2 and 3). For assessing each tooth, the pairs were given three minutes. The teeth were examined visual-tactilely by a ballpoint probe and a 3-in-1 air syringe under the light of the dental unit in a simulation laboratory. They used DIAGNOdent pen® on only three teeth. The device was calibrated by each student according to the manufacturer’s directions on a ceramic standard block and individually on a caries free surface before scanning each tooth.

### Scoring

The most severe site of the occlusal caries lesion of 24 teeth was registered. The students gave scores using ICDAS score (0–6) (Table 2), activity assessment by modified Nyvad’s method (0–6) (Table 3), and. traditional **clinical assessment, CE (0 = sound, 1 = inactive initial caries, 2 = active initial caries, 3 = dentine caries)**, The students assessed additionally three teeth using CE (0–3) and DIAGNOdent pen® scanning, scoring the peak value (0–99) of the occlusal surface.

No instructions were given about the order of scoring, but the students were allowed to ask about the criteria and the use of the DIAGNOdent pen® device.

### Validation

#### Clinical validation for Nyvad’s method and (CE)

Before the workshop digital photographs had been taken of all the teeth. Also, all 27 teeth were assessed visual-tactilely using the traditional clinical (CE) and lesion activity assessment (Nyvad) by the supervisors (HP and VA). HP and VA used in their assessment a ballpoint probe, light of the unit, a magnifying glass, and digital photographs. The obtained scores were used as a clinical golden standard for the traditional clinical (CE) and Nyvad scores by the students.

#### Histological validation for ICDAS and LF

After the students completed the examination, each tooth was cut vertically into two sections aiming to expose the most severe carious site by a Horico Superdiaflex Diamond Disc® 0.15 mm mounted in KaVo EWL K9 (25,000/min) handpiece (*KaVo* Dental GmbH, Biberach, Germany). The sectioned tooth halves were photographed and the depth of caries lesions was assessed by HP and VA from the magnified digital photographs on a computer monitor. The depth of enamel demineralization was assessed and recorded according to the deepest extension of the demineralization. The depth of dentine demineralization was assessed and recorded according to depth of the color change compared to the surrounding sound tissue. After forming a common agreement on lesion depth of each tooth in the manner described above, the supervisors gave teeth histological scores (Table 1). which were used as a golden standard for the students’ ICDAS and LF scores.

### Statistical analyses

For the analyses, the ICDAS scores were re-coded into four categories as follows; 0➔0, 1➔1, 2➔2, 3&4➔3, 5&6➔4, to make them comparable with the histological golden standard scores (Tables 1 and 2). The LF scores were categorized according to the manufacturer’s guidelines (0–13 ➔1, sound; 14–20 ➔2, enamel caries; 21–29 ➔3, deep enamel caries; >30 ➔4, dentine caries). The categorized scores were compared with the histological golden standard (Table 1). To evaluate the correlation between the LF values and histological lesion depths, Spearman’s correlation coefficient (ƥ) was calculated for all three teeth.

To evaluate the validity of the ICDAS method, mean sensitivity and specificity values (SD) were calculalted for each tooth (dentine caries D3 vs. sound tooth). Sensitivity was calculated using the cut-off point ICDAS values 3–6 and histological score 3–4 (lesion depth to middle dentine). To evaluate inter-examiner agreement, weighted κ (SD) **values** and **intra-class correlation coefficient (ICC, 95% CI) values were calculated considering all lesions.** The data were analyzed using SPSS (version 16.0, SPSS, Inc., Chicago, Il, USA).

## Results and discussion

### Results

Sectioning of the teeth revealed that two out of the 27 teeth were caries free, 5 had caries extending histologically into the inner half of the enamel or outer third of the dentine, 9 into the middle third of the dentine, and 10 into the inner third of the dentine.

**Figure 1:**
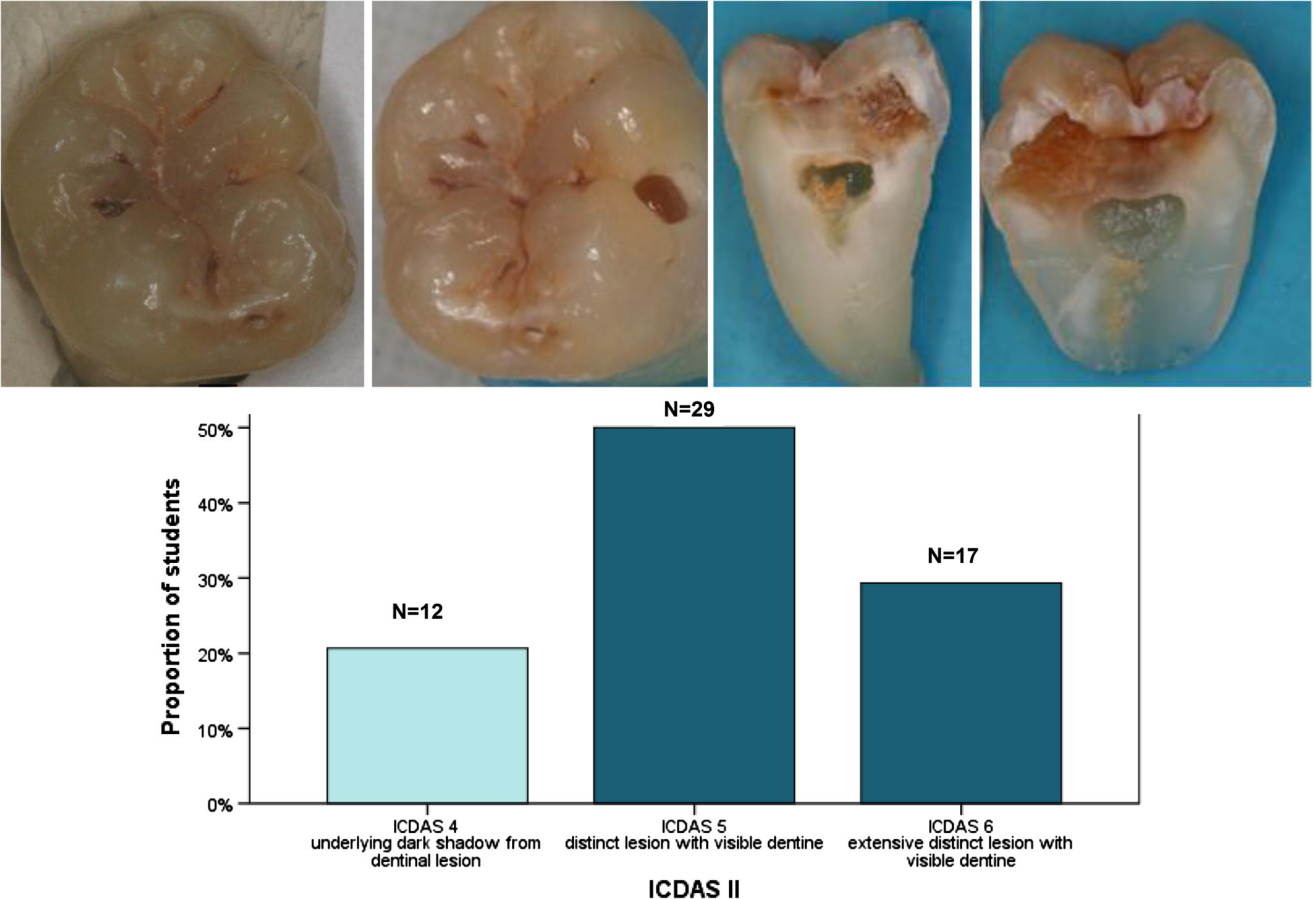
**An example of a caries lesion which collected mostly correct scores by the third-year dental students, validated with a histological golden standard.** Distribution of the dental students’ estimation of the ICDAS score is presented; histological score is presented in a darker color. During the workshop, probing by the students turned the micro cavities into true ones (picture in the middle).

**Figure 2:**
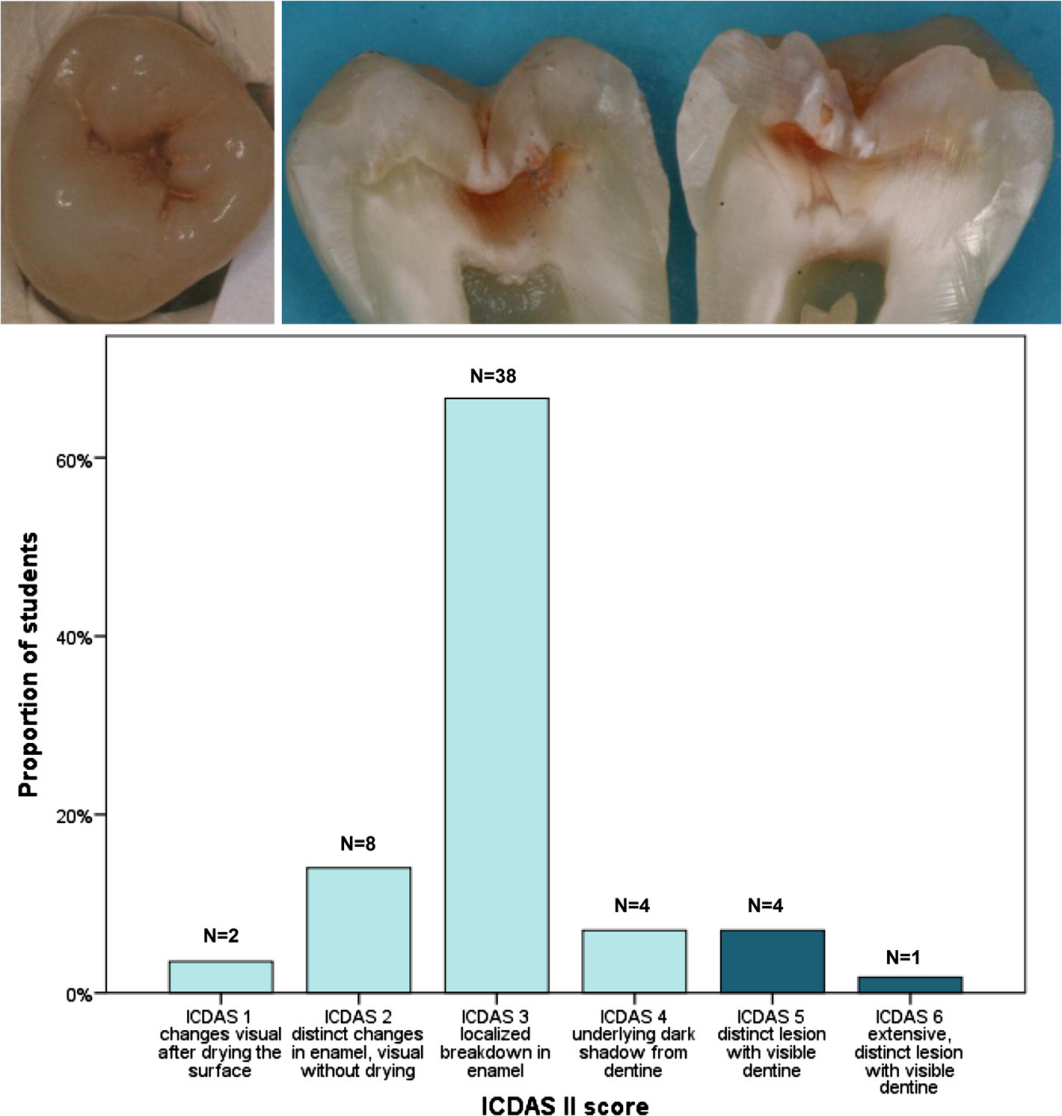
**An example of a caries lesion which surprised the students by its true histological depth.** Distribution of the dental students’ estimation of the ICDAS score is presented; histological score is presented in a darker color.

The sensitivity and specificity values for the ICDAS scores at level D3 were very good, 0.78 (0.12) and 0.87 (0.13) respectively. The κ and ICC values considering all lesions of different lesion depth, suggested fair to good accuracy of the performance among the students for all of the assessed methods (Table 4). Evaluating lesions correctly was the easiest for the students concerning teeth with the most severe lesions (Figure 1), whereas in the distribution of scores of initial lesions the range was wide (Figure 2).

**Table 4:** Intra-examiner agreement with golden standard using different caries detection methods by the third-year dental students expressed as kappa (κ) and intra-class correlation coefficient (ICC) values

**Assessment system**	**κ Mean (SD)**	**ICC mean (95% CI)**	**Validation**
Clinical	0.53 (0.101)	0.69 (0.65-0.70)	Clinical
Nyvad	0.48 (0.087)	0.68 (0.66-0.71)	Clinical
ICDAS	0.47 (0.094)	0.66 (0.64-0.69)	Histological

DIAGNOdent pen® values of the sound tooth agreed 100% with the histological appearance (median 3; min 0, max 7). In the case where caries extended into the inner third of dentine, the agreement was 78% (median 99; min 73, max 99). When caries histologically extended into the middle third of dentine, the DIAGNOdent pen® values had a wide range (median 24; min 5, max 88); only 25.5% of the values fell within the category >30, dentinal caries according to the manufacturer. These findings were supported by the Spearman’s correlation coefficient; ƥ was 1.000 when the tooth was distinctly decayed, 0.302 when the tooth was sound (p = 0.021), and 0.240 when the tooth was moderately decayed (p = 0.240).

### Discussion

Caries risk assessment, diagnosis and synthesis is considered as one of the five main domains in cariology, and the base of clinical decision-making [3]. Particularly the assessment of activity of lesions is essential in selecting between non-operative and operative treatment. Reliable caries detection and lesion activity assessment are cornerstones in turning the philosophy from the operative treatment schema in today’s clinical practice to non-operative caries control schema in the future [3]. It is essential for all dentists to know the new schema to be applied in their clinical practice. This schema as well as caries detection ad lesion activity assessment must be emphasized in dental curriculum. Combining lectures and hands-on workshops as a teaching method of caries detection is one of the possibilities recommended by the European Core Curriculum [16] but until this study, it had not been used in Finland. This work investigated how easily caries detection is adopted by dental students, and how accurate their performance is using different methods after basic training. According to the present results, third-year dental students are very well capable of embracing different caries detection and lesion activity assessement methods when introduced by lectures and a hands-on workshop. Our findings are in concordance with other studies supporting the capability of efficient learning of caries detection methods even by beginners [1,8,10,17].

It was the easiest for the students to evaluate lesions correctly in the most severe cases. The lesions that were difficult for the students to score (ICDAS) could have surprised even an experienced examiner by their depth. The accuracy of their decisions using ICDAS method (dentine caries D3, as the cut off point) was very good. Inter-examiner agreement concerning lesion depth and activity of all lesions was fair to good using all methods. In calculating sensitivity and specificity (accuracy) D3 was used as cut off point, whereas all scores were considered in evaluating inter-examiner agreement. The findings are in line with other studies reporting good accuracy and inter-examiner agreement both by Nyvad’s and ICDAS methods [18-21], even if some of those studies were carried out on primary teeth. Methods using classifications are easily introduced and adapted, are accurate and reproducible, which will hopefully make them popular among clinicians. To our knowledge they are mainly used in research, so far.

Validation i.e. “golden standard” is a major issue in evaluating methods for caries detection and lesion activity assessment. Validating lesion depth detection in *in vitro* studies can be accomplished reliably by sectioning the observed teeth and estimating lesion depth using magnification (microscope, magnifying glasses, computer screen). Using PC screen has been shown to be a valid and applicable method comprapble with the findings through a microscope [22]. Validation can also be radiographical considering its limitations especially in detecting occlusal lesions and secondary caries lesions. In *in vivo* studies and studies estimating lesion activity, validation is subjective, done by a reference examiner i.e. “golden standard”. The histological validation was carried out in this study in evaluating the outcome by the ICDAS and LF methods whereas lesion activity assessment was validated clinically by reference scores (Nyvad’s method and CE). It was emphasized to the students that estimating and evaluating lesion depth reliably on extracted teeth is difficult if possible at all. However, every dentist must be aware of signs indicating lesion depth and activity of the lesions. Gimenez et al. [10] reported that use of prescriptive methods (like ICDAS, or Nyvad’s method) is easier for undergraduate students whereas adopting such new approaches (classification of lesions) is challenging for experienced colleagues, who rely on their experience i.e. their approach is heuristic, prone to bias.

Nyvad’s system is based on the dynamic nature of caries, when environmental changes may affect de-remineralization balance, causing detectable consequences [12] even in weeks, either as progress or arrest of the lesions. In this study, a modified version of Nyvad’s method was used, i.e. the lesions were categorized according to the continuity of the surface to three categories, and activity was assessed in each category. Estimating signs of activity on extracted teth is not fully comparable with a clinical situation. This may explain confusion of the students when estimating lesion activity. The results showed, however, good agreement on estimation of activity of the lesions by the students according to clinical validation by the supervisors, which again supports the validity of the method [12]. In the respective works on dental students and staff by Zandona et al. [9] and Gimenez et al. [10], all groups were equally capable of evaluating lesion activity either using descriptive or prescriptive methods. However, activity evaluation using clinical signs was quicker to perform than the one with scoring (LAA) [10]. Authentic clinical situation is always easier for evaluating lesion activity which was emphasized to the students of the present study.

To describe reliability for detecting and assessing different kinds of lesions by all methods and by all examiners, ICC and kappa values were calclulated. The proportion of fully sound teeth and teeth with initial lesions in our sample was smaller compared to the situation in the clinical practice. This most likely influences the outcome in the present study, as it is easier for the dentist to diagnose a tooth sound than to find caries lesions. In the present study, on the other hand, the teeth with most severe lesions were correctly diagnosed.

Working as pairs may be considered a factor causing some bias in the present study; pairs often had exactly same scores (5 pairs of the total 28 pairs) even though they had been told to determine the scores individually. Another factor distorting our results may have been the use of probes by the students. On the photographs taken before and after the workshop, we could notice some micro cavities having turned into true ones during the workshop. The reason for that might have been the difficulty in determining lesion activity on extracted tooth surfaces and the fact that the soft surface wears away easily after multiple probing. This change of surface continuity might have caused some variation in the scores on lesion depth. Today, probing of suspected lesions is recommended to be carried out by feeling tactilely the surface structure. For students, it should be emphasized that probing does not promote sensitivity of the caries detection, but may cause irreversible tooth damage [23].

The students were allowed to question about the criteria and use of DIAGNOdent Pen, this might have influenced the results. This was done, however, to support the students in a situation with the time limit and facing many new criteria simultaneously. Again, it can be speculated, if sequential decisions on the same tooth was a source of bias. Even if the students were just learning the criteria it is possible that some quick learners could adopt the respective scores by different methods which may have influenced the results.

To eliminate the cross infection, the storing solution for the extracted teeth had been denatured alcohol. *In vitro*, of course, makes estimating lesion activity more difficult that *in vivo*. Additionally, compared with, for example, freezing, storing in alcohol may have had some effect on the tooth surface and therefore complicate accurate diagnostics [24]. The inter-examiner agreement was not affected by this. However, it might have affected laser fluorescence measurements because fluorescence is believed to be caused by bacterial metabolic porphyrins in caries lesions [25].

Sectioning of the teeth in two halves was done to reveal the most severe-looking site on the occlusal surface. In magnified digital photographs, the evaluation of the depth of the lesions was unproblematic [20]. On the other hand, it can be discussed if the most representative site of the lesion was reached by the free-hand sectioning of the lesion, considering also that cutting destroys a small part of the lesion. This could have caused some bias in the histological scores or golden standard. The optimal way would have been chemical fixation and cutting the teeth in several very thin slices and examining them with more accurate histopathological techniques [26]. For the learning/teaching process, teeth should be sectioned immediately after the hands-on session and should be available at the feedback lecture to demonstrate lesion depth and activity. However, this was not possible here.

Histological agreement with the DIAGNOdent pen® scores by the students was excellent on the fully intact teeth and on the teeth with an extensive caries lesion. When caries extended into the middle third of dentine, the range of the scores was wide, reported also elsewhere [27]. The performance of the students using DIAGNOdent pen® scanning in the present study is in concordance with the literature [28]. However, the results on LF scanning shall be considered with great precaution due to the limited number of the teeth (n = 3) evaluated.

## Conclusions

Accuracy and reproducibility of different caries detection and lesion activity assessment methods seems to be good among the third-year dental students without previous diagnostic experience even after basic training only. Combining lectures with a hands-on practice can be considered as a good method for introducing dental students to caries detection using classification of the lesions as well as lassessing lesion activity. With new caries treatment schema, teaching new caries detection methods is essential for future dentists. Education must be continuously up-dated and evaluated.

## Competing interests

The authors declare that they have no competing interests.

## Authors’ contributions

VA is the designer, supervisor and conductor of this project, HP, a fourth year dental student at the time of the field phase of the study, participated in the project in all its phases (design, implementing, writing). HV’s biggest contribution is statistical, he also participated in the writing process. LT gave his scientific expertise to the project. M-LL has actively participated in writing the paper. All authors read and approved the final manuscript.

## Pre-publication history

The pre-publication history for this paper can be accessed here:

http://www.biomedcentral.com/1472-6831/13/70/prepub
